# Correction: A microbial survey of the International Space Station (ISS)

**DOI:** 10.7717/peerj.4029/correction-1

**Published:** 2018-02-27

**Authors:** Jenna M. Lang, David A. Coil, Russell Y. Neches, Wendy E. Brown, Darlene Cavalier, Mark Severance, Jarrad T. Hampton-Marcell, Jack A. Gilbert, Jonathan A. Eisen

**Affiliations:** 1 Genome Center, University of California, Davis, CA, United States of America; 2 Science Cheerleader, United States of America; 3 The Consortium for Science, Policy & Outcomes, Arizona State University, Tempe, AZ, United States of America; 4 Scistarter.org, United States of America; 5 Biosciences Division, Argonne National Laboratory, Lemont, IL, United States of America; 6 Department of Biological Sciences, University of Illinois at Chicago, Chicago, IL, United States of America; 7 Argonne National Laboratory, University of Chicago, Lemont, IL, United States of America; 8 Institute for Genomics and Systems Biology, Argonne National Laboratory, Lemont, IL, United States of America; 9 Evolution and Ecology, University of California Davis, CA, United States of America; 10 Medical Microbiology and Immunology, University of California, Davis, CA, United States of America; 11 Biomedical Engineering, University of California, Davis, CA, United States of America

Corrigendum:

After publication, we noted terminological issues with the use of rRNA genes and OTUs in the article in addition to typographical issues in the Methods section, Reference list and Acknowledgements. 

In the “DNA extraction and library preparation” section of the Methods, we have removed “-htp” from the first sentence. In the same paragraph, we have replaced the lowercase u in “ul” with the Greek letter mu. 

Terminology issues with rDNA genes and rDNA:

The terminology we used to describe surveys of 16S rRNA genes was sometimes inappropriate (we sometimes referred to “16S rDNA genes”, which do not exist) and also now recommended against, even though it is technically correct (16S rDNA). Some of this was due to simple editing errors (16S rDNA genes) and some of it was using terminology we have previously used but which is now recommended against (16S rDNA). See https://microbiomejournal.biomedcentral.com/submission-guidelines/preparing-your-manuscript/research-article for some recommended guidelines about this we were not aware of. We therefore have changed all usage to “16S rRNA genes”. None of these affect the meaning in any way.

Terminology regarding OTUs vs. species:

Additionally, our study made use of common methods to classify sequences into what are known as “OTUs” or operational taxonomic units. We stated that these are used as a proxy for species but did not intend to say “species” in parts of the paper when we meant OTUs. In the Methods and Results and Discussion sections, we wrote that OTUs were a proxy for species for people who were not familiar with OTUs. It was pointed out to us by multiple people that this just confuses the matter and therefore we have therefore removed all uses of “species” when we mean “OTUs”. We have also removed the “proxy” lines. Some of these changes may affect how people interpret our text (if they thought we were truly quantifying species) but do not in any way impact the conclusions of our work. 

Reference issues:

There a number of issues in the references. We have updated the references to add a DOI for Aas et al. (2005); Arndt et al. (2012); Belzer & De Vos (2012); Cogen, Nizet & Gallo (2008); Grice et al. (2009); Knief et al. (2010); Krych et al. (2014); Langille et al. (2014); Linton & Hinton (1988); Liu, Tan & Exley (2015); Murphy et al. (2007); Ribeiro et al. (2011); Schwarzberg et al. (2014); Seifried, Wichels & Gerdts (2015); Steyn et al. (1998); Willems (2014); Young et al. (2007) and Zaura et al. (2009). We have also corrected the titles of Brooks et al. (2015), Maule et al. (2009), McDonald et al. (2011), Novikova et al. (2006) and Venkateswaran et al. (2014). Ichijo et al. (2016) was published with the incorrect issue name (“April”); this has been removed. McMurdie & Susan (2013) has been corrected to McMurdie & Holmes (2013). The author list of Menninger et al. (2013) has been corrected to Dunn et al. (2013); we have also corrected the title. The author list of Caporaso (2012) has been corrected to Caporaso et al. (2012); we have also corrected the title and journal name in addition to adding the volume and pagination information. In-text citations to McMurdie & Susan (2013), Menninger et al. (2013) and Caporaso (2012) have been updated to reflect the correct author list. We have also added a new reference to Wickham (2009) to the reference list and replaced the reference to Wilkinson (2011) on page 7 with this new reference.

Acknowledgement Section:

There is a spelling error for a name (Steven Kimball) in the Acknowledgements and the ORCID link / ID is incorrect. We have corrected this to read Steven Kembel and the ORCID ID to be: 0000-0001-5224-0952.

